# A Systematic Review of the Recent Quality of Life Studies in Adult Extremity Sarcoma Survivors

**DOI:** 10.1155/2012/171342

**Published:** 2012-08-23

**Authors:** Melissa H. Tang, Donald J. W. Pan, David J. Castle, Peter F. M. Choong

**Affiliations:** ^1^Department of Orthopaedics, St Vincent's Hospital Melbourne, Level 3 Daly Wing, 35 Victoria Parade, Fitzroy, VIC 3065, Australia; ^2^Faculty of Medicine, Dentistry and Health Sciences, The University of Melbourne, VIC 3010, Australia; ^3^Engage 1 on 1 Psychology, 181 High Street, Northcote, VIC 3070, Australia; ^4^Department of Psychiatry, St Vincent's Hospital Melbourne, 41 Victoria Parade, Fitzroy, VIC 3065, Australia

## Abstract

*Background*. Extremity sarcoma represents a heterogeneous group of rare cancers that carries a relatively high morbidity with regards to physical function. Quality of Life (QoL) as an outcome is an important consideration in this cohort. We aimed to identify the correlates of QoL in extremity sarcoma cohorts. *Methods*. A systematic review of the literature on extremity sarcoma in adults from five databases over the last ten years was undertaken. *Results*. Twelve articles were chosen and assessed for quality. Physical and social function of extremity sarcoma survivors is below that of the general population. Overall QoL scores of these patients are comparable to those of the general population. Studies that used more recently treated cohorts found that patients who had limb sparing surgery displayed superior functional outcomes over those that underwent amputations. Pain and perceiving that the cancer negatively influenced opportunities was associated with poor outcomes. *Conclusion*. The available literature regarding QoL in extremity sarcoma patients is heterogeneous in terms of aims and assessment tools. Results need to be interpreted in light of the improved management of extremity sarcoma in more recent patient cohorts.

## 1. Introduction

Extremity bone and soft tissue sarcoma represents a rare and heterogeneous group of bone and connective tissue cancers, which accounts for 0.2% and 0.5% of all cancers in Australia [1], respectively. Most musculoskeletal neoplasms occur within specific age ranges and have a predilection for specific sites. 60% of all sarcomas occur in people who are younger than 55 years old [2]. The arbitrary cutoff for the definition of adolescents and young adults (AYA) is not clearly defined in the literature; however, most studies take the limit of fifteen years old [3]. Adolescence represents a period of developmental transitions, characterised by cognitive, biological, and socioeconomic challenges [4]. Health problems such as cancer, in this age group, are uncommon as cancer is predominantly a disease of the older population. Considering cancer in all age groups, sarcoma is rare. In Australia, soft tissue sarcoma was the 9th most common cancer, accounting for 3.2% (279 cases) of all cancers in AYA between 2003 and 2007. During this timeframe, bone cancer was the second most common cause of cancer death (107 deaths, 10.5% of all cancer deaths) whilst soft tissue cancer was the seventh most common cause of cancer death (60 deaths, 5.9% of all cancer deaths) in AYAs. The mortality and morbidity associated with sarcoma is high. Paediatric cancer centres and adult cancer centres may not be adequately equipped to manage the unique demands of cancer patients in this age group [5]. In Victoria, we manage patients who are older than fifteen years old in an adult cancer facility.

Advancements in medical imaging technology, greater understanding of the biology of tumours, development of powerful chemotherapeutic regimens, improvements in technique, and availability of reliable reconstruction options have revolutionised the management of sarcoma and resulted in improved mortality and morbidity of sarcoma survivors.

Neoadjuvant therapy has made it possible for some patients with previously unresectable tumours to undergo limb sparing surgery through tumour regression [6]. It is recognised that there is no difference between limb sparing surgery and amputation in terms of survival or local recurrence, provided adequate margins are obtained and, therefore, limb sparing surgery is the gold-standard surgical option for a patient with extremity sarcoma, especially for distal lower limb tumours [7–10]. Research using more recently treated cohorts found that physical function was superior following limb sparing surgery compared to amputation [8, 11].

It is recognised that the level of physical functioning of sarcoma survivors is below that of the general population. Despite that, the QoL of survivors appears to remain comparable to that of the general population.

Health-related quality of life (QoL) is a multidimensional construct that considers the impact of an individual's health status on his or her life and opportunities. It is subjective and may be modified by impairments, functional status, perceptions, and social opportunities and may be influenced by disease, injury, treatment, and policy [12, 13]. Given that QoL has to be considered in the context of the individual's environment; QoL studies should be interpreted in the context of the timeframe it was performed. QoL studies that were performed more than a decade ago may represent a different clinical and social environment compared to more recently performed studies. Various studies have reported trends towards improved outcomes with time [14–16]. Apart from improved chemotherapeutic regiments, these authors have postulated that this is due to the formation of multidisciplinary sarcoma groups. In Victoria, the Victorian Cooperative Oncology Group, Sarcoma Section was established in 2006. We have chosen an arbitrary timeframe of QoL studies performed in the last decade to represent studies that are more relevant to the current clinical and sociopolitical environment.

Measures of QoL are divided into three main domains: physical function, mental health, and social well being [13].

To explore the issue of QoL in extremity sarcoma survivors that is relevant to our practice, we performed a systematic review of journal articles published in the last decade on QoL in sarcoma in studies that assessed patients who were fifteen years or older. The key question guiding OUR review was the following: what are the modulators of QoL in extremity sarcoma? The aims of our study was to (1) evaluate the quality of the current literature on QoL in extremity sarcoma, (2) identify the tools used to assess QoL in extremity sarcoma, (3) evaluate the physical function, mental health and overall QoL of extremity sarcoma survivors compared to the general population, (4) compare the functional and QoL outcomes between limb sparing surgery and amputation, and (5) identify the factors that are postulated to influence QoL outcomes (determinants of QoL).

## 2. Search Strategy and Criteria

We performed a systematic review of the current literature from five databases (PubMed, CINAHL, Psychinfo, Medline, and Melbourne University Library Catalogue) with the following keywords: sarcoma AND “quality of life”.

Exclusions included: articles published before 2001, haematological malignancies, Kaposi sarcoma, retinoblastoma, paediatric sarcomas (mean age at diagnosis (MAD) <15 years old, or mean age at surgery <15 years old if MAD not provided), QoL studies on heterogeneous, general cancer population, QoL of other noncancer populations in general, basic sciences research or animal model studies, and nonextremity sarcomas. We also excluded case reports and articles for which full text was not available as well as articles in which physical function alone was assessed and other domains of QoL were not assessed. Non-English articles with English translations were included.

## 3. Results

A total of 3713 articles were found. The reviewers (M. H. Tang and D. J. W. Pan) independently applied exclusion criteria and removed duplicates through reading titles and abstracts. Nine articles were found that met inclusion criteria. Other relevant articles were tracked through systematic reviews and a further three studies were included (total twelve).

### 3.1. Results: Quality Assessment

We utilized the Newcastle-Ottawa Quality Assessment Scale (NOS) for cohort studies to assess the quality of the articles [28]. The NOS awards stars for three categories: “Selection”, “Comparability”, and “Outcome”, each divided into further subcategories. Each study can be awarded a maximum of one star for each subcategory whilst Comparability can be awarded a maximum of two stars as summarized by Table 1.

#### 3.1.1. Selection

- Representativeness: we awarded one star if the study clearly described the aims of the study and set about to clearly achieve the aims with the appropriate cohort chosen.
- Selection of the nonexposed cohort: we awarded one star if the comparative group, for example, amputees versus limb sparing surgery, was derived from the same community as the entire extremity sarcoma group.
- Ascertainment of exposure: we awarded one star if the authors noted confirmation of the diagnosis of extremity sarcoma via medical records.
- Demonstration that outcome of interest were not present at the start of the study: we awarded one star if baseline function and QoL was noted.

#### 3.1.2. Comparability

- We awarded one star if the study controlled for era of management. We defined this to be the entire cohort operated on in a ten-year timeframe. A further star was awarded if the study controlled for anatomical location, type of sarcoma, or grade of sarcoma.

#### 3.1.3. Outcome

- Assessment of outcome: we awarded one star if the assessment tool was a well-known and widely used validated tool, for example, SF-36, EORTC QLQ C-30, TESS, MSTS87, and MSTS93 (abbreviations under Table 2).
- Duration of followup: we awarded one star if the mean followup was one year or longer.
- Adequacy of followup of cohorts: We awarded one star if there was more than 70% response rate OR if the nonresponders were statistically analysed to account for sampling bias.

#### 3.1.4. Sample Size

From the selected articles, two studies had a participant size of more than 200 [11, 19], six had between 100 and 200 participants [18, 21, 22, 25–27], none had between 50 and 100 participants and four had between 20 and 50 responders [17, 20, 23, 24].

#### 3.1.5. Study Design

Of all the studies, nine were retrospective studies [11, 17, 19–21, 23, 25–27], whilst two were longitudinal studies [18, 22].

#### 3.1.6. Cohort-Anatomical Location

Six studies looked at a specific anatomical location. Out of these, four looked at tumours involving the lower limb [11, 17, 19, 21], whilst one study looked at tumours around the knee [20].

#### 3.1.7. Cohort-Histological Subtypes

Some studies looked at specific types of bone or soft tissue sarcoma subtypes. For example, two studies examined osteosarcoma and Ewing's sarcoma patients [17, 25]. Some studies studied a heterogeneous cohort of bone sarcoma patients [20] or soft tissue sarcoma patients [18, 22, 23, 26].

#### 3.1.8. Aims

Two studies were performed with a specified aim of descriptive statistics purposes. They described the functional, QoL, and/or oncological outcomes within their defined cohort [25, 27]. Most studies, however, included a description of their cohort when measuring their outcomes.

Two studies were performed to compare QoL between patients that had undergone limb sparing surgery and those that had an amputation [19, 21]. Eiser et al. [17] included a third group—those that had an amputation following failed limb sparing surgery. Three groups compared limb sparing and amputation according to resection level [11, 19, 25].

One study was performed with a sole aim to identify the prevalence of depression and anxiety throughout different phases of the disease [27]. Five studies stated prevalence of psychological distress in their cohort [11, 19, 23, 24, 27].

Five studies compared their findings against published figures for the reference normal population [17, 18, 20, 23, 25].

Six studies attempted to identify determinants of QoL [17, 21–23, 25, 26]. For example, Schreiber et al. [22] aimed to evaluate how functional disabilities impacted on QoL, whilst Davidge et al. [26] attempted to evaluate how preoperative expectations impacted upon postoperative function and QoL. Thijssens et al. [23] looked at determinants of QoL and posttraumatic stress symptoms in their cohort of locally advanced soft tissue sarcoma patients who had all undergone neoadjuvant chemotherapy.

One study compared QoL and functional outcomes between patients who had neoadjuvant radiation therapy prior to limb sparing surgery and those that had adjuvant radiation therapy following limb sparing surgery [18].

#### 3.1.9. Tools Used

Table 2 summarizes the tools used by each study. The most widely used tools were the TESS, MSTS87, and SF-36. Specific groups tended to use similar tools. For example, the German authors tended to use the FLZ [21], whilst the Canadian group tended to include TESS, and if MSTS was used, that tended to use the 1987 version [18, 22, 26]. The Americans used their own computer-generated tool [11, 19].

### 3.2. Study Design and Measure

Study design and measures used were heterogeneous as summarized in Table 3.

### 3.3. Results: Findings

#### 3.3.1. Comparing the Sarcoma Population with the General Population

Studies utilizing global QoL assessment tools found that physical and social function for sarcoma survivors was significantly lower than that of the general population when measured with the SF-36 [17, 18, 23]. In these studies, emotional functioning and mental health scores were not significantly different between sarcoma survivors and the general population. 

#### 3.3.2. Comparing Type of Surgeries: Limb Sparing Surgery versus Amputation

Six studies were performed to compare QoL and physical function between limb sparing surgery and amputation [11, 17, 19, 21, 23, 25]. The studies are summarised in Table 4. 

Using an arbitrary cutoff of 1990, studies that used cohorts treated after 1990 were able to show that limb sparing surgery displayed significantly better functional outcomes compared to amputation [17, 21, 23, 25]. Studies that used cohorts treated before 1990 failed to show a significant difference [11, 19]. 

#### 3.3.3. Identification of Determinants of Quality of Life

Despite poor physical function, sarcoma survivors reported high scores on QoL assessments, with scores comparable to that of the general population [17, 21, 25]. Therefore, physical function may not be the biggest discriminant of QoL in extremity sarcoma survivors. Other determinants may contribute to satisfactory QoL as summarized in Table 5. Various studies attempted to examine the different determinants of well being, which may evolve in the face of cancer. In a study of long-term survivors of sarcoma, 94% of participants stated that they felt that the cancer had made them a “better person” [24].

**Specific Determinants of QoL**

Four studies evaluated the ability of chosen variables to predict QoL.

Other factors involved in one or more aspects of QoL were studied and described below.

**(i) Sport Participation Following Surgery**

Only about half the population studied by Pardasaney et al. [11] reported being active in sport, whilst Refaat et al. [19] found that only one patient participated in a contact-type sport. Both studies did not correlate sport participation with QoL. Aksnes et al. [25] found that physical activity per se was not significantly associated with poor functional outcomes; however, it was not assessed if a change in baseline physical activity was significant.

**(ii) Employment**

Employment was studied in four studies as shown in Table 6. The most consistent finding in these studies is that in a subset of patients, cancer negatively influenced work or school and this was significantly associated with poor outcomes.

**(iii) Personality**

Three studies attempted to study some facet of personality as shown in Table 7. Being optimistic was consistently associated with good function outcomes.

**(v) Psychological Distress**

Mental health is one of the domains of QoL and is therefore assessed by QoL assessment tools.  Table 8 summarises the findings regarding mental health by studies that used QoL tools. A further three studies assessed mental health directly by utilizing tools designed specifically for the detection of psychological distress. The results of these three studies are summarized in Table 9. In general, studies that used QoL tools found that mental health scores were comparable to that of the general population. However, studies that used other measures of psychological distress revealed that a significant proportion of the cohorts studied displayed significant psychological distress.

**(vi) Tumour Characteristics**

Davis et al. [18] found that patients who had a larger resection specimen and those who had motor nerve sacrifice did proportionately worse than baseline in terms of function. Recurrence and higher-grade tumour at diagnosis were associated with poorer psychological and functional outcomes [23, 27]. Pain also significantly correlated with poor outcomes [25] and conversely, experiences that involved little pain were associated with good outcomes [23]. A summary of the findings form the selected studies is shown in Table 10.

## 4. Discussion 

We performed a systematic review of twelve recently published articles that looked at QoL in grown-up patients with extremity sarcoma. We chose a cut-off age of fifteen years old, as this is the cutoff in Victorian hospitals for patients to be treated in an adult facility. 

The available literature regarding QoL in extremity sarcoma patients is heterogeneous in terms of aims and assessment tools. The most widely used tools were the TESS and MSTS87 for physical function assessment and the SF-36 for global QoL assessment. 

Our review confirmed that physical functioning, pain, and social functioning of extremity sarcoma survivors are significantly worse than the general population [17, 18, 23]. 

Despite poorer function, overall QoL appears to be comparable to that of the general population. In a study of long-term survivors of sarcoma, 94% of participants stated that they felt that the cancer had made them a “better person” [24]. We were interested in the mechanism underlying the process of evolving and building resilience to adversity. The concept of “response shift” has been described by Sprangers and Schwartz to describe an evolution of internal standards, values, and conceptualisations in the face of disability such as that produced by cancer that influences one's self-perception of QoL [29]. The concept of “response shift” has been documented as a coping strategy in the general cancer literature. Hence, it is possible that survivors of extremity sarcoma adapt to their physical limitations and “grow” from their experiences with cancer; physical function is less important than other factors, such as other psychosocial variables as described above, in determining their QoL. 

Regarding the mental health component of QoL, there is a mismatch in findings. Studies that use QoL assessment tools have found that emotional health domains are similar between sarcoma survivors and the general population. However, when studies used tools designed to detect psychological distress, the prevalence of distress in the cohorts ranged from 13.7% to 30.8% for depression and 11.8% to 29.2% for anxiety and 12% for PTSD. 

In the Australian wider community, the prevalence of anxiety disorders is reported to be 14.4%, of which the prevalence of PTSD is reported to be 6.4%. The reported prevalence of depression in the general population is 6.2% [30]. 

Sarcoma can affect not only the life but also the livelihood of those it afflicts. Job security and the ability to perform work tasks may be impaired. The time required for management and recuperation, with imposed or consequent restrictions may lead to loss of ability to contribute to the workforce. The ability to work has personal economic, psychological, and community benefits. Weiner et al. [24] found that survivors of sarcoma who were unemployed were more likely to display psychological distress compared to those who were employed. Studies that looked at employment status in survivors of sarcoma failed to consider the unemployment rate in the general population during the timeframe of the study. Other studies on employment could usefully investigate the obstacles to finding gainful employment following surgery for extremity sarcoma. For example, pain has been determined to be a major limiting factor in obtaining gainful employment [23]. This may reflect that being occupied is a good distraction for pain, or that pain is a significantly limiting factor for engaging in work-related activities. Nevertheless, it highlights the importance of good pain management, with a multidisciplinary, multimodal approach to pain management. Apart from work, further longitudinal research into change from baseline sport and recreational activity participation, including level of participation, is important concerning determinants of QoL. 

Personality facets influence perception of health and QoL. Cognitive interpretations of the locus of control are influenced by the diagnosis of cancer, which interacts with personality factors, socioeconomic status, depression, and anxiety [31]. Few studies had investigated the role of personality in influencing outcomes in extremity sarcoma patients. 

Our review identified that there is a significant prevalence of psychological distress in extremity sarcoma survivors. However, few studies have attempted specifically to correlate psychological distress with QoL. 

Compared to the normal population, people with chronic disease and cancer such as sarcoma frequently interact with healthcare professionals as part of their followup. These sessions present opportunities for screening, detection and management of psychological distress. Certain groups may be at risk of psychological distress that are not detected and “fall through the gaps” and, hence, not be managed effectively. This may account for the near equal prevalence of current psychological distress symptoms in sarcoma patients and the normal population where overt depression and anxiety are detected and managed. No study reported the incidence of treated psychological distress premorbidly or during treatment for the sarcoma. Paredes et al.'s [27] finding that the prevalence of psychological distress wanes with time highlights the need for longitudinal research to determine protective factors that enhance resilience in sarcoma patients. Psychological distress has not been thoroughly investigated but appears to have an important but poorly defined effect on outcomes in patients treated surgically for their sarcoma. Apart from directly affecting QoL, psychological distress may jeopardise management through negative health behaviours, poor decision-making processes, and ineffective rehabilitation to regain optimal mobility. Psychological distress is a modifiable factor through recognition and effective management strategies. Further research into the detection and management of mental health needs of patients with sarcoma is thus important to improve overall outcomes. 

There is no gold standard measure of QoL for extremity sarcoma. The MSTS87, MSTS 93, and TESS assess for subjective physical function and objective functional impairment only and does not consider other aspects of QoL that of emotional and social domains. The SF-36 was most commonly used to assess QoL. It consists of eight equally weighted scales of QoL assessment. It is a generic subjective QoL tool that does not take into consideration the unique considerations that are relevant to the extremity sarcoma cohort [32]. For example, it may not take into account the implications of sarcoma as cancer, which may have implications on a threat to life and symptoms of therapy (such as nausea and vomiting during chemotherapy). The EORTC QLQ C-30 may also be inappropriate for use in extremity sarcoma as high scores during nontreatment times may be elevated as it assesses symptoms such as nausea, vomiting, and change in bowel habits. However, it assesses financial difficulties, which may be relevant to the extremity sarcoma cohort, given that patients may experience adverse influences with regards to career opportunities. 

From our review, we found that different variable differentially influenced QoL. We found that pain, activity restriction, and a perception that the cancer negatively influenced opportunities were associated with poor QoL. 

There is, thus, a need to identify the most discriminatory determinants of QoL in extremity sarcoma patients to develop an extremity sarcoma specific QoL tool for accurate measurement of QoL in this unique cohort. Such a tool will have clinical application in the standardisation of clinical research that compares different treatment modalities. 

## 5. Conclusion 

Advancements in technology has revolutionised the management of sarcoma and markedly improved the mortality and morbidity of sarcoma. This paper has identified directions for future research, the need for the development of an extremity-sarcoma specific and age appropriate QoL tool, and assessment of modulating biological, psychological, and social factors that influence coping. We propose that a longitudinal, prospective design would best fit these research needs. There is also emerging evidence to suggest that psychological distress is an important but often overlooked aspect of care. This justifies further research into the assessment of the role of psychological distress in influencing overall outcomes following surgery for extremity sarcoma. We feel that interventional strategies, such as mindfulness training to reduce the level of psychological distress to enhance QoL outcomes following sarcoma surgery, are a worthwhile endeavour, given its promise in other cancers [33]. 

## Figures and Tables

**Table 1:** Quality assessment of selected studies.

Reference	Date of publication	NOS rating		
		Selection	Comparability	Outcome
Eiser [17]	2001	∗∗∗	∗	∗∗∗
Davis et al. [18]	2002	∗∗∗∗	∗∗	∗∗∗
Refaat et al. [19]	2002	∗∗	∗	∗∗∗
Rödl et al. [20]	2002	∗∗∗		∗∗
Zahlten-Hinguranage et al. [21]	2004	∗∗	∗	∗∗
Pardasaney et al. [11]	2006	∗∗∗	∗	∗
Schreiber et al. [22]	2006	∗∗∗∗	∗∗	∗∗∗
Thijssens et al. [23]	2006	∗∗∗	∗	∗∗∗
Weiner et al. [24]	2006	∗∗∗	∗	∗∗∗
Aksnes et al. [25]	2008	∗∗∗	∗	∗∗∗
Davidge et al. [26]	2009	∗∗∗	∗∗	∗∗∗
Paredes et al. [27]	2011	∗∗∗	∗	∗∗

**Table 2:** Tools used in the assessment of QoL in sarcoma patients.

Tool	Number of studies	References
MSTS87	3	Schreiber et al. 2006 [22], Davidge et al. 2009 [26], Davis et al. 2002 [18]
MSTS93	2	Zahlten-Hinguranage et al. 2004 [21], Aksnes et al. 2008 [25]
TESS	4	Eiser et al. 2001 [17], Schreiber et al. 2006 [22], Davidge et al. 2009 [26], Davis et al. 2002 [18]
SF-36	4	Eiser et al. 2001 [17], Aksnes et al. 2008 [25], Thijssens et al. 2006 [23] (used Dutch version of RAND-36), Davis et al. 2002 [18]
EORTC QLQ C-30	2	Zahlten-Hinguranage et al. 2004 [21], Rödl et al. 2002 [20]
Semistructured interviews	2	Weiner et al. 2006 [24], Eiser et al. 2001 [17]
BSI	1	Weiner et al. 2006 [24]
IES	2	Weiner et al. 2006 [24], Thijssens et al. 2006 [23]
Body image instrument	1	Eiser et al. 2001 [17]
Computer generated 5 page 87 question tool	2	Refaat et al. 2002 [19], Pardasaney et al. 2006 [11]
FLZ	1	Zahlten-Hinguranage et al. 2004 [21]
HADS	1	Paredes et al. 2010 [27]
RNL	2	Schreiber et al. 2006 [22], Davidge et al. 2009 [26]
EQVAS	2	Schreiber et al. 2006 [22], Davidge et al. 2009 [26]
LOT	2	Schreiber et al. 2006 [22], Davidge et al. 2009 [26]
Fatigue questionnaire	1	Aksnes et al. 2008 [25]
Single item, questions	N/A	Aksnes et al. 2008 [25]: pain, stiffness, influence of sarcoma on choice of career and level attained, and activity participation (hours per week) differentiated into low and high energy demand activity type, Davidge et al. 2009 [26]: socioeconomic questions Thijssens et al. 2006 [23]: perception of involvement in decision making and satisfaction based on a 5-point Likert scale
Outcome expectation self-report questionnaire	1	Davidge et al. 2009 [26]: length of hospitalization, complications, difficulty with daily activities

MSTS87: Musculoskeletal Tumour Society Score 1987 version; MSTS93: Musculoskeletal Tumour Society Score 1993 version; TESS: Toronto Extremity Salvage Score; SF-36: Short Form 36 Health Survey; EORTC QLQ C-30: European Organisation for Research and Treatment of Cancer Quality of Life Questionnaire Core Module 30; BSI: Brief Symptom Inventory; IES: Impact of Event Scale; FLZ: Freiburger Life-Contentment List; HADS: The Hospital Anxiety and Depression Scale; RNL: Reintegration to Normal Living Scale; EQ-VAS: EuroQoL Visual Analogue Scale; LOT: Life Orientation Test.

**Table 3:** Summary of study design.

Reference	Study design/sample	Measures used	Timeframe of treatment
Eiser 2001 [17]	Retrospective cross-sectional study of patients with osteosarcoma and Ewing's sarcoma of the lower limb *N* = 37 Median age of diagnosis = 19 (7–37) years Median time since diagnosis = 10 (2–33) years	SF-36 Body image instrument TESS: daily competence Use of analgesia and gait aids Semistructured interview	1977–1995

Davis et al. 2002 [18]	Prospective randomized study of nonmetastatic extremity STS patients Tumour size was dichotomized at 10 cm, then randomized to PreRT (*n* = 88) or PostRT (*n* = 94) *N* = 182 MAD = 54.7 (18.8–93.8) years	MSTS87 TESS: primary measure: 10-point difference considered significant SF-36 Timepoints: baseline (at randomisation), 6 weeks, 3, 6, 12, and 24 months after surgery	1994–1997

Refaat et al. 2002 [19]	Retrospective study on patients with lower limb sarcoma *N* = 408; Amp *n* = 66, LSS *n* = 342 MAD = 47.5 years	Computer generated questionnaire	1972–1987

Rödl et al. 2002 [20]	Patients with high grade malignant bone tumours of distal femur that underwent rotationplasty *N* = 22 Mean followup = 12 (10–18) years Median age at time of study = 28 (18–49) years	EORTC QLQ C-30FLZ Education level	

Zahlten-Hinguranage et al. 2004 [21]	Retrospective, cross-sectional study of patients with lower limb (excluding foot and ankle) sarcoma *N* = 124 (LSS *n* = 102; Amp *n* = 22) Mean age at assessment = 35 (14–76) years Mean time from surgery = 46 (6–250) months	EORTC QLQ-C30 MSTS93 FLZ	1980–2000

Pardasaney et al. 2006 [11]	Retrospective comparative study on patients with sarcoma of the lower limb with at least 2-year followup *N* = 408 (Amp *n* = 65, LSS *n* = 343) Mean age at surgery = 40.36 (2–86) years Mean time from treatment = 8.91 (2–27) years.	Computer generated quality of life questionnaire	

Schreiber et al. 2006 [22]	Longitudinal study on nonmetastatic STS patients who had LSS *N* = 100 MAD = 55 (18–86) years	MSTS87 TESS RNL EQ-VAS LOT 2 timepoints: preoperatively and 1 year postoperatively	2001–2003

Thijssens et al. 2006 [23]	Retrospective cross-sectional study on survivors of locally advanced, nonmetastatic STS, who underwent isolated limb perfusion, with intentional delayed LSS *N* = 39 (LSS *n* = 30, Amp *n* = 9) Median age at perfusion = 49 (14–72) years Median time since perfusion = 7 (1–13) years	IES SF-36 Perception of involvement in decision Treatment satisfaction: 5-point Likert scale	1991–2003

Weiner et al. 2006 [24]	Cross-sectional study of long-term survivors of sarcoma *N* = 34 MAD = 16 (7–34 years) Mean age at study = 34 (17–54 years) Mean time from diagnosis = 18 (4–33 years)	Semi-structured interview BSI (intensity of psychological distress) IES	

Aksnes et al. 2008 [25]	Retrospective study on patients with osteosarcoma or Ewing's sarcoma *N* = 118 (LSS *n* = 67, Amp *n* = 51) MAD = 18 (2–44) years Mean time since diagnosis = 13 (6–22) years	SF-36 TESS MSTS93	1982–2000

Davidge et al. 2009 [26]	Retrospective cohort study on adult patients with nonmetastatic extremity STS who underwent LSS Outcome measures assessed at two time points: preoperatively and 12 months postoperatively Categories collapsed to allow for sufficient powering *N* = 157	Outcome expectation questionnaire MSTS87 TESS RNL EQ5D-VAS LOT	2001–2005

Paredes et al. 2011 [27]	Cross-sectional descriptive study on sarcoma patients in different phases of disease *N* = 142 Dx *n* = 42, mean time from diagnosis = 4.27 months; Rx *n* = 37, mean time from diagnosis = 10.94 months, mean time from treatment = 9.34 months; Fx *n* = 63, mean time since completion of treatment = 52.93 months MAD = 48.32 years	HADS Demographic and clinical questionnaire	

STS: soft tissue sarcoma; PreRT: neoadjuvant radiation therapy; PostRT: adjuvant radiation therapy; MAD: mean age of diagnosis; Amp: amputation; LSS: limb sparing surgery; Dx: diagnosis phase; Rx: treatment phase (1st treatment, whether it was chemotherapy, radiation therapy, and/or surgery counted as index timepoint); Fx: followup phase.

**Table 4:** Summary of studies comparing limb sparing surgery with amputation.

Author	Timeframe that cohorts were treated	Conclusions regarding QoL	Conclusions regarding physical function
Eiser 2001 [17]	1977–1995	No significant difference	LSS superior over amputation
Refaat et al. 2002 [19]	1972–1987	No significant difference	No significant difference
Zahlten-Hinguranage et al. 2004 [21]	1980–2000	No significant difference	LSS superior over amputation
Pardasaney et al. 2006 [11]	—	No significant difference except for above knee amputation, which had inferior outcomes	No significant difference except for above knee amputation, which had inferior outcomes
Thijssens et al. 2006 [23]	1991–2003	No significant difference	LSS superior over amputation
Aksnes et al. 2008 [25]	1982–2000	No significant difference	LSS superior over amputation

**Table 5:** Studies that correlated variables with overall QoL.

Reference	Finding
Eiser et al. 2001 [17]	Low TESS scores and poor body image predicts poor QoL
Schreiber et al. 2006 [22]	Complications of surgery associated with poor QoL; low TESS scores predicted poor QoL
Davidge et al. 2009 [26]	Uncertain expectations associated with poor QoL
Thijssens et al. 2006 [23]	Pain associated with poor QoL

**Table 6:** Summary of employment characteristics.

Reference	Correlation of unemployment with outcomes	Unemployment rate	Further findings
Aksnes et al. 2008 [25]	Unemployment not significantly associated with poor functional outcomes	(i) 11% of cohort unemployed (ii) 50% of cohort reported that cancer had negatively influenced employment or educational choices	(i) “Employment and education opportunities negatively influenced by cancer” significantly associated with poor functional and QoL outcomes (ii) Less than one-third of cohort involved in physically demanding job

Pardasaney et al. 2006 [11]	Not assessed	(i) Patients with below-knee tumours: 47.4% amputees and 23.3% LSS patients unemployed (ii) Patients with above knee tumours: 42.9% of amputees and 33.6% of LSS patients unemployed	

Thijssens et al. 2006 [23]	Not assessed	11.1% of patients of working age were unemployed	

Weiner et al. 2006 [24]	Unemployment significantly associated with psychological distress	(i) 35% of cohort unemployed (ii) 24% of cohort reported having trouble with keeping up job or school requirements	“Difficulty with keeping up requirements” significantly associated with psychological distress

**Table 7:** Summary of personality characteristics.

Reference	Personality proxy measure	Correlation with outcomes
Schreiber et al. 2006 [22]	Optimism (LOT)	Optimism significantly negatively correlated with poor physical function but no significant association found with QoL

Thijssens et al. 2006 [23]	(i) Locus of control (ii) Satisfaction with treatment	(i) “Perception of having less involvement in decision making process for treatment” significantly associated with psychological distress (ii) Patients who had outcomes consonant with preoperative expectations reported more satisfaction postoperatively (iii) Dissatisfaction with treatment was associated with psychological distress

Davidge et al. 2009 [26]	(i) Optimism (LOT) (ii) Expectations of outcome	(i) Optimism predictive of good functional and QoL outcomes (ii) Pessimism associated with having uncertain expectations regarding recovery following surgery

**Table 8:** Summary of mental health in extremity sarcoma patients—a reflection from QoL tools.

Reference	QoL tool	Results
Eiser et al. 2001 [17]	SF-36	Mental health subscale comparable to general population
Davis et al. 2002 [18]	SF-36	Mental health subscale comparable to general population but role emotional lower than general population
Refaat et al. 2002 [19]	Self-reported depression or anxiety	Prevalence in cohort of depression (17–26%), anxiety (22–26%)
Rödl et al. 2002 [20]	EORTC QLQ C-30	Mental health subscale comparable to general population
Zahlten-Hinguranage et al. 2004 [21]	EORTC QLQ C-30	Not displayed
Pardasaney et al. 2006 [11]	Self-reported depression or anxiety	Patients who had above knee amputations were at an increased risk of developing anxiety; otherwise, comparable to general population; prevalence in LSS: depression (30.8%), anxiety (29.2%); prevalence in amputation cohort: depression (17.6%), anxiety (11.8%)
Aksnes et al. 2006 [14]	SF-36	Poor functional subscales significantly correlated with poor emotional role functioning

**Table 9:** Summary of mental health in extremity sarcoma patients—a reflection from IES, BSI (GSI), and HADS.

Reference	Tool	Results
Thijssens et al. 2006 [23]	IES RAND-36	Prevalence of PTSD in cohort: 12.2%; RAND-36 emotional subscale scores were comparable to reference population

Weiner et al. 2006 [24]	IES BSI	77% of cohort displayed psychological distress

Paredes et al. 2011 [27]	HADS (distress represented by moderate to severe scores)	Overall prevalence of anxiety: 24.6%, depression: 13.7%

**Table 10:** Summary of study aims and findings.

Reference	Aims	Findings and conclusions
Eiser 2001 [17]	(1) Compare QoL of sample to normal population (from previously published data), (2) compare QoL in LSS versus primary (1o) Amp versus secondary (2o) Amp (amputation following failed LSS), (3) qualitatively assess decision making and adaptation to 2o Amp, (4) Identify determinants of QoL	*Comparing against normal population*. QoL for sarcoma survivors significantly lower (*P* < 0.05); emotional functioning and mental health scores equal; physical function, physical role function, and social function very significantly different (*P* < 0.001). *Comparing LSS versus Amp*. QoL equal; LSS less likely to use gait aids; LSS better “daily competence” (TESS), which is more clearly associated with QoL than compared to Amp. Authors conclude that this is because LSS have higher expectations, whilst Amp more resigned to limitations. Daily competence was inversely proportional to age and males reported better physical functioning compared to females. *Qualitative study on 2o Amp*. 80% did not regret initial LSS and felt that was time gained to allow psychological adaptation to diagnosis and subsequent surgery. This group was associated with reporting that they were involved in decision-making process regarding the decision to amputate, that is, they had a sense of control. Conversely, the group who were not satisfied reported that they had no input into decision to amputate that is, they lacked a sense of control over the situation.

Davis et al. 2002 [26]	(1) Evaluate function and QoL in patients with extremity STS, comparing PreRT (higher complication rate) versus PostRT (higher likelihood of fibrosis) for wound complication outcomes, (2) To compare cohort's SF-36 scores with that of the general population	*Comparing RT timing*. PreRT more likely to have wound complications requiring intervention (*P* = 0.01). Otherwise, timing of radiotherapy has no significant impact on function. *Trajectory of rehabilitation*. Decrease in function from baseline up to 6 weeks, gradual increase up to 6 months. Mean scores returned to baseline at 1 year. SF-36 followed above trend, except for social function, role emotional, and mental health subscales that gradually improved over time, with mean scores at 2-year mark being higher than baseline. *Comparing to general population*. Physical function, role physical, and general health significantly lower at all timepoints. Vitality social and mental health subscales approximated the referenced normal population at the 2-year mark. Mental health significantly lower up to 6 month postoperation mark. *Tumour characteristics as determinants of outcome*. MSTS change scores negatively predicted by lower limb tumour, large resection specimen, and motor nerve sacrifice TESS, SF-36, and bodily pain change scores negatively predicted by lower limb tumour and prior incomplete excision. Wound complications had the largest association with low MSTS and TESS scores.

Refaat et al. 2002 [19]	Compare LSS versus Amp. Specific items analysed. ability to ambulate, climb stairs, drive, employment, sports participation Anxiety, drug dependence, depression, sleep problems, limitation of sexual performance, number of children, menstrual problems	*Gait and mobility*. LSS more likely to report a limp (*P* < 0.04). The more proximal the surgery, the more likely the patients required gait aids. A third of patients found difficulty with stairs. *Drive*. Almost all patients able to drive, independent of surgery. *Sport*. Males more likely to engage in sports than females but sport participation rate independent of surgery. No contact sports except 1 (kickboxing). *Employment and social function*. Males more likely than females to be employed and within males, employment more likely in LSS than Amp (*P* < 0.04). Marriage in LSS = Amp. Menstrual irregularities in LSS > Amp (*P* < 0.0006; 89% of women) (perhaps secondary to chemotherapy). Active sex life Amp > LSS (*P* < 0.04; Amp. 88%, LSS 75%). Amp > LSS to have children (*P* < 0.00004; Amp. 52%, LSS. 19%). Erectile dysfunction independent of surgery.
		*Mental health*. 26% of Amp, 17% of LSS reported feeling periodically depressed. 26% of Amp, 22% of LSS reported anxiety. LSS = Amp for sleep disturbance, analgesic requirements, satisfaction, physical function, psychosocial outcomes, and use of gait aids. *Overall satisfaction*. longer followup correlated with more satisfaction.

Rödl et al. 2002 [20]	Assess QoL and socioeconomic status versus normal population	No decrease in psychosocial adaptation and life contentment compared to normal population (significance values not presented). 12 patients (54.5%) stated that the operation had not affected the choice of profession. Rotationplasty patients were less contented with job and income but, otherwise, no significant difference. This suggests that rotationplasty is superior over amputation if LSS it not possible (however, it did not actually compare with amputation).

Zahlten-Hinguranage et al. 2004 [21]	(1) Compare QoL of LSS versus Amp, (2) identify discriminants of Qol	QoL in LSS = Amp, MSTS scores better with LSS versus Amp; worse with increasing anatomical level of surgery (though significance not reported). *Determinants of QoL*. LSS. physical performance status with sports and recreational activities (the authors hypothesize that this is because in LSS the disability may not be apparent and, therefore, participation restriction and reintegration plays a key role in determinants of QoL); Amp: social acceptability (the authors hypothesize that this is due to the visible mutilation, there is a greater emphasis on facets of relationships and social support network) and good self-reported health.

Skaliczki 2005	Correlate function and QoL to type of resection, length of resection, type of prosthesis, and tumour site	No correlation between functional outcome and QoL with type of implant and length of resected bone. Patients with tumour in distal femur had significantly better functional and QoL outcomes than those with proximal tibial sarcoma. All patients displayed at least “good” emotional acceptance (“Enthusiastic” *n* = 13) Most common complication was infection (11%).

Pardasaney et al. 2006 [11]	Compare long-term physical function and psychological outcome between LSS versus Amp at 4 different anatomical levels (groups). Below knee (BK), above knee (AK), and hip and pelvis	*Prevalence of depression and anxiety*. BK group—depression. 17.6% (Amp) and 30.8% (LSS); anxiety. 11.8% (Amp) and 29.2% (LSS). AK group—depression. 40.9% (Amp) and 17.4% (LSS). *Prevalence of unemployment and no participation in sport*. BK group—unemployment. 47.4% (Amp) and 23.3% (LSS); no sport participation. 55% (Amp) and 46.7% (LSS). AK group—unemployment. 42.9% (Amp) and 33.6% (LSS); no sport participation. 61.3% (Amp), 60% (LSS). *Comparing LSS and Amp*. Equal functional and psychological outcomes at BK level. At AK level, anxiety in Amp > LSS 2.4 times (*P* = 0.021); Amp > LSS for limp (*P* = 0.005), use of walking aid (*P* = 0.004) and ability to drive (0.05). Amp < LSS in terms of subjective muscle weakness (0/009). Amp = LSS for perception of good health, depression, and cosmetic deformity. The more proximal the level of LSS, the more subjective weakness was likely to be reported (*P* < 0.001), the more likely was a limp reported (*P* = 0.011) and the less likely was a prosthetic used (0.033).

Schreiber et al. 2006 [22]	Evaluate how functional disability impacts on HRQoL at 1-year postoperative mark by assigning tools to the categories of (1) impairment (MSTS87), (2) activity limitation (TESS), and (3) participation restriction (RNL), using HRQoL as the outcome (EQ-VAS). Sample was adjusted for demographic and clinical variables and subject to further analysis	Mean MSTS = 30.5 (=87.3%), TESS = 88.4, RNL = 97.7, EQ-VAS = 80.5; reexcision rate = 37%. All tools correlated with each other. *Determinants of QoL*. when unadjusted for demographic and clinical variables, for example, complications (wound or nerve/vessel—not graded), MSTS most impact on EQ-VAS. When adjusted. gender significantly impacted on participation restriction; complications impacted on activity limitation and participation and restriction. MSTS not significantly associated with EQ-VAS; RNL (participation restriction) was only factor that was significantly associated with EQ-VAS. Concluded that whilst impairment and activity limitations affect activities of daily living, restriction in participation of life roles and situations (e.g., employment or leisure activities) has the greatest effect on HRQoL No association found between radiotherapy (not specified if neoadjuvant or adjuvant) and functional outcome. *Personality factors on self-reported QoL*. Level of optimism did not significantly predict QoL.

Thijssens et al. 2006 [23]	(1) QoL in STS survivors versus normal population (age matched 2006 Dutch population); (2) identify determinants of QoL and posttraumatic stress symptoms (PTSS)	*Comparing to normal population*. STS < normal for physical function (*P* < 0.001) and role limitation due to physical problems (*P* = 0.01). Employment associated with less pain (*P* = 0.14). Educational level and having a partner not related to QoL or PTSS. *Amp versus LSS*. Amp < LSS for physical and social function (*P* = 0.016, *P* = 0.023), more role limitations due to physical and emotional problems (*P* = 0.017, *P* = 0.014) Otherwise, Amp = LSS. *Prevalence of PTSS*. No Amp patients displayed clinically significant PTSS compared to 26.7% of LSS patients (20.5% of total cohort). *Role of tumour characteristics*. Patients who developed metastases had worse physical functioning (*P* = 0.034), more role limitations due to physical (*P* = 0.032) and emotional (*P* = 0.004) problems. *Role of involvement in decision making process*. Patients who indicated that choice of treatment was dictated by the surgeon only showed significantly decreased social functioning (*P* = 0.057), more role limitation and intrusion (*P* = 0.23). Higher satisfaction significantly associated with less intrusion (*P* < 0.0001) and lower IES scores (*P* < 0.0001), with better social functioning (*P* = 0.024), more vitality (*P* = 0.046), and better health perception (*P* = 0.028). *Determinants of satisfaction*. (1) Matching of preoperative expectation with postoperative outcomes, (2) outcomes better than expected, or (3) Absence of pain.

Weiner et al. 2006 [24]	(1) Identify prevalence of psychological distress and PTSS in long-term survivors, (2) compare prevalence of psychological distress with that in the normal reference population according to published data in 1993	77% of the cohort displayed significant psychological distress. 12% of the cohort met criteria for PTSD. IES correlated with GSI. *Male gender = risk factor for untreated psychological distress*. Compared to women, men more likely to display significant psychological distress, have higher IES and avoidance scores (*P* < 0.001, *P* < 0.05, 0 < 0.01, resp.). Compared to normal population, men had higher GSI scores and displayed more intrusion (*P* < 0.05, *P* < 0.001). GSI scores similar to psychiatric patients. *Reintegration and its association with long-term psychological distress*. 24% of participants reported trouble with keeping up with school/job requirements following treatment. They scored higher on GSI, PST, and avoidance (all *P* < 0.05). *Employment*. 65% employment rate. Unemployed people more likely to score higher on GSI (*P* < 0.05). *Effect of tumour characteristics on mental health*. Recurrence showed a trend towards higher GSI scores (86% versus 56%). Age, stage of disease, and time since diagnosis were independent of psychological distress. *Cognitive adaptation*. 94% of participants stated that they felt they were a better person today as a result of the experience of sarcoma. *Social adaptation*. Patients who had difficulty maintaining friendships during treatment reported more intrusion (*P* < 0.05) and avoidance (*P* < 0.05).

Aksnes et al. 2008 [25]	(1) Evaluate long-term functional outcome following surgery for osteosarcoma or Ewing's sarcoma. (2) Identification of determinants of QoL, for example, by examining if impaired function influenced QoL and ability to work. (3) Compare QoL and psychological distress with normal population	Median MSTS = 70% (17–100%); Amp < LSS; *P* < 0.001; median TESS = 89% (43–100%); LSS = Amp; *P* = 0.34. *Resection level*. Above knee amputees lower MSTS and TESS; *P* = 0.003, *P* = 0.02, respectively, compared to below knee tumours. *Comparing Amp with LSS*. Amp = LSS except for physical functioning, bodily pain, and physical component summary scale. *Correlates with MSTS ≤ 50%*. Lower scores in physical components of SF-36 (*P* < 0.05) and emotional role functioning (*P* = 0.03). *Employment*. 31% involved in a physically demanding job. Half of the patients believed that the cancer had influenced their choice of education or job. Controlling for age at diagnosis and type of surgical treatment, these people had poorer MSTS, TESS, and SF-36 scores (*P* < 0.001 and *P* = 0.001, resp.). *Activity participation*. 84% considered physically active. LSS = Amp and independent of MSTS score. Chronic muscular pain or stiffness people more likely to be physically inactive; *P* = 0.004.

Davidge et al. 2009 [26]	(1) To examine the impact of preoperative outcome expectations on postoperative function and QoL. (2) To identify determinants of outcome expectations	Complications (Grade 3 and above) occurred in 23%, of which, 91% were wound-related complications. *Role of expectations*. A significant proportion of people did not know what to expect regarding length of recovery (28%), complications (27%), and difficulties with daily activities (20%). Uncertain expectations were associated with poorer outcome (*P* < 0.05) Pessimism and low educational attainment were associated with uncertain outcomes This may be mediated through self-efficacy beliefs versus underlying cognitive processing underpinning the two versus external confounders, for example, psychological distress affecting both.

Paredes et al. 2011 [27]	(1) Describe prevalence of depression and anxiety according to phase of treatment in a cross-sectional study, (2) identify determinants of adjustments in different phases of disease	*Majority of patients had limb sparing surgery * . 23.8% in treatment group and 8.3% in follow-up group had amputation. Moderate to severe anxiety most prevalent in the Dx (29.3%), followed by Rx (25%) and Fx (21.3%). Difference not significant between groups. Patients diagnosed for a longer time and patients who terminated treatments for a longer time had showed lower anxiety levels (perhaps reflecting that the emotional distress is normal transitory and allows for adjustment). Moderate to severe depression most prevalent in Rx (19.4%)—females had an increased depression score, followed by Diagnosis phase (19%) and Follow-up phase (6.6%). Difference was not significant between groups. Older patients had higher levels of depression (perhaps reflecting other stressors), whilst depressive symptoms abated with time since treatment. *Determinants of psychological distress*. Dx—presenting status (*P* = 0.01 for depression, *P* = 0.04 for anxiety); recurrence of disease (*P* < 0.05 for anxiety, *P* < 0.05 for depression). Rx—females more likely to have depression (*P* < 0.5). Fx—time since diagnosis and time since completion of treatment were negatively correlated with anxiety (*P* < 0.5, *P* < 0.5, resp.), whilst older age and less time since completion were associated with depression (*P* < 0.05, *P* < 0.05, resp.).
